# Seasonal energy exchange in sea ice retreat regions contributes to differences in projected Arctic warming

**DOI:** 10.1038/s41467-018-07061-9

**Published:** 2018-11-27

**Authors:** Robyn C. Boeke, Patrick C. Taylor

**Affiliations:** 1 Science Systems Applications Inc, Hampton, VA 23666 USA; 2 NASA Langley Research Center, Climate Science Branch, Hampton, VA 23681-2199 USA

## Abstract

Rapid and, in many cases, unprecedented Arctic climate changes are having far-reaching impacts on natural and human systems. Despite state-of-the-art climate models capturing the rapid nature of Arctic climate change, termed Arctic amplification, they significantly disagree on its magnitude. Using a regional, process-oriented surface energy budget analysis, we argue that differences in seasonal energy exchanges in sea ice retreat regions via increased absorption and storage of sunlight in summer and increased upward surface turbulent fluxes in fall/winter contribute to the inter-model spread. Models able to more widely disperse energy drawn from the surface in sea ice retreat regions warm more, suggesting that differences in the local Arctic atmospheric circulation response contribute to the inter-model spread. We find that the principle mechanisms driving the inter-model spread in Arctic amplification operate locally on regional scales, requiring an improved understanding of atmosphere-ocean-sea ice interactions in sea ice retreat regions to reduce the spread.

## Abstract (web summary)

State-of-the-art climate models disagree on the magnitude of Arctic climate change. Here the authors show that the principle processes driving the disagreement operate locally in sea ice retreat regions, necessitating comprehensive observations to improve our understanding of atmosphere-ocean-sea ice energy exchanges.

## Introduction

The Arctic has warmed 2–3 times faster than globally-averaged warming, a phenomenon known as Arctic amplification (AA)^1,2^. AA is evident in surface temperature observations over the last century^3,4^, in model projections made starting in the 1970’s^5,6^ and suggested by Arrhenius^7^ more than 100 years ago. Arctic climate change has global consequences by influencing glacial melt and sea level rise, permafrost thaw and the carbon cycle, atmospheric and oceanic circulations, and potentially extreme mid-latitude weather^8,9^. Because of the significant implications for the physical climate and human systems, accurate projections of AA are needed. Unfortunately, climate models project a wide range of possible futures for the Arctic—a larger inter-model spread than any other region. The 2 °C globally-averaged warming specified in the Paris Climate Agreement equates to an Arctic warming between 3 and 7 °C, according to Coupled Model Intercomparison 5 (CMIP5^10^) models. Narrowing the inter-model spread requires an understanding of how Arctic feedback processes and their interactions shape the temperature response.

A collection of interacting processes support AA: sea ice loss and surface albedo feedback^2,5,7,11–16^, changes in longwave and/or temperature feedbacks^17,18^, cloud changes^6,19–21^, intraseasonal cycling of heat^22,23^, and poleward energy transport^24–27^. While the surface albedo feedback (SAF) has often been cited as the leading contributor to AA^28^, idealized climate simulations show that AA can occur in its absence^29,30^. Recent studies argue for a remote forcing of observed AA, whereby atmospheric heat transport into the Arctic from lower latitudes drives warming and thinner sea ice^31,32^, whereas others attribute observed AA to local mechanisms such as the surface albedo and evaporation feedbacks^11^. Our understanding of AA mechanisms has evolved significantly over the last decade, yet the relative importance of each feedback and its contributions to the inter-model spread in Arctic warming projections remains under debate.

This study offers a seasonal, process-oriented surface energy budget decomposition using the multi-model CMIP5 archive and methods introduced in Lu and Cai^17^. CMIP5 models show an increased inter-model spread in surface temperature and sea ice compared to CMIP3^33^. Pithan and Mauritsen^18^ argue that temperature feedbacks explain these inter-model differences, however our analysis and interpretation differ. Previous studies have analyzed AA in CMIP5 models, yet generally lack the regional perspective required to isolate the energy exchanges that appear to regulate AA in observations^34^. Moreover, CMIP5 models exhibit large differences in the seasonal cycles of radiative fluxes, clouds, and turbulent fluxes^15,35,36^ and a seasonality in the inter-model spread in projected warming.

 Our results outline the primary drivers of inter-model spread in AA found in CMIP5 models using a surface energy budget perspective highlighting the important contribution of seasonal energy exchange in sea ice retreat regions facilitated by ocean heat storage to the inter-model spread in Arctic warming. We argue that the atmospheric and ocean processes that modulate the seasonal energy exchange in sea ice retreat regions drive model differences in projected Arctic warming. The models that more effectively disperse energy drawn from the surface in sea ice retreat regions warm more. Therefore, reconciling the differences in AA projections requires constraining the representation of atmosphere-ocean-sea ice interactions in sea ice retreat regions.

## Results

### Model projections of Arctic amplification

**Fig. 1:**
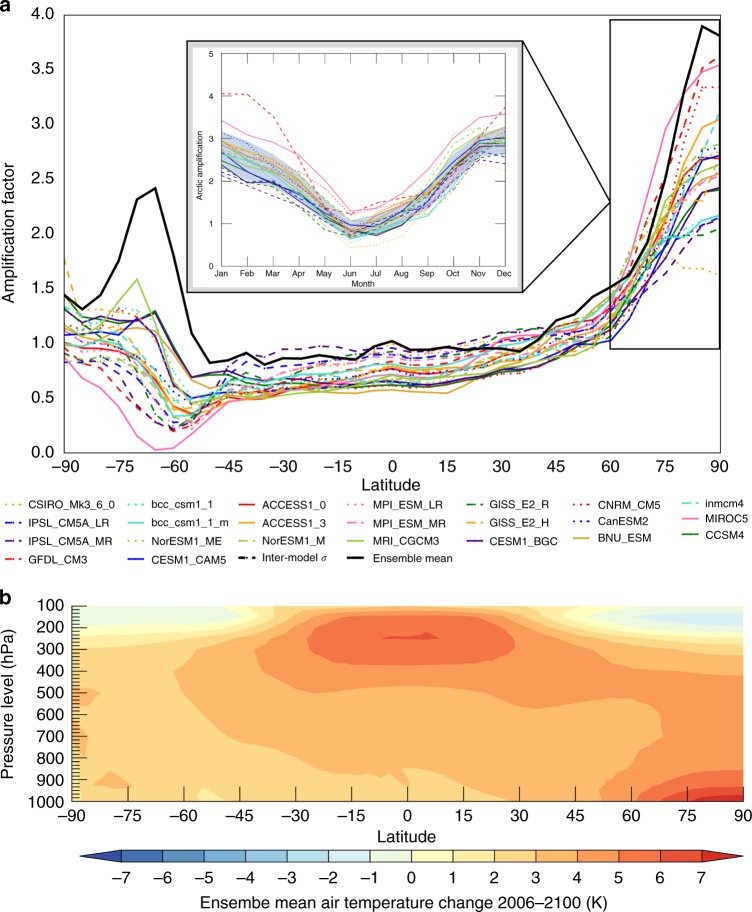
Nature of projected Arctic amplification. Surface temperature amplification for CMIP5 RCP8.5 models. Figure 1a shows the zonally-averaged temperature change normalized to global temperature change for each CMIP5 model (hereafter amplification factor). The solid black line in **a** represents the ensemble mean amplification factor; the black dashed line represents the zonally-averaged inter-model standard deviation normalized by the global inter-model standard deviation. The boxed region in **a** represents the Arctic domain 60–90°N used in this study. The inset in **a** shows the seasonal cycle of the amplification factor for the Arctic domain. **b** shows the vertical profile of ensemble mean temperature change by latitude

The current generation of CMIP5 climate models unanimously simulate AA in response to increasing CO_2_ (Fig. 1). Figure 1 illustrates AA using the normalized temperature change—hereafter, amplification factor—defined as the ratio between the 1° zonally-averaged temperature change to global temperature change. All models simulate surface-based warming—at least 1.5 times global average warming—extending into the lower troposphere poleward of 75°N (Fig. 1b). The solid black line in Fig. 1a represents the ensemble mean amplification factor, which exceeds 2.5 at the pole. CMIP5 models also simulate a similar seasonality of AA (Fig. 1a, inset) with minimum warming (amplification factor < 1) in summer and maximum warming in fall and winter (amplification factor >2). Despite unanimous agreement in the existence of AA, models disagree on the magnitude and spatial characteristics.

Inter-model differences in projected Arctic warming exceed those for any other latitude. The Arctic domain (defined as 60°–90°N) shows increasing model differences at more northerly latitudes, approaching an amplification factor spread of 1.6–3.6 (Fig. 1a). The dashed black line in Fig. 1a represents the ratio between the 1° zonally-averaged inter-model standard deviation in temperature change to the global inter-model standard deviation—a measure of inter-model spread. This ratio approaches four times the global average moving poleward. The seasonal cycle of AA (Fig. 1a, inset) shows the largest model spread in winter (amplification factor between 2 and 4) and smallest in summer (amplification factor between 0.5 and 1.25).

**Fig. 2:**
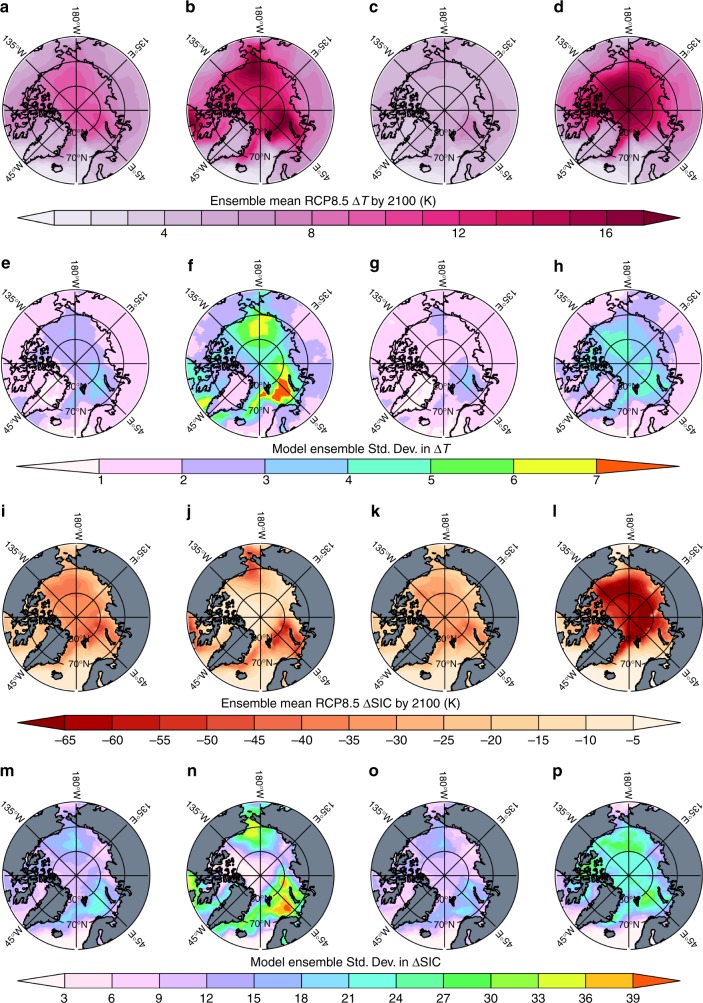
Ensemble mean and standard deviation of Arctic climate change by 2100. CMIP5 RCP8.5 projected surface temperature change by 2100 for (**a**) annual mean, (**b**) winter (January–February), (**c**) sunlit season (March through September), and (**d**) autumn (October, November, and December) with the corresponding ensemble standard deviations (**e**–**h**). **i**–**l** show the ensemble mean projected changes in sea ice concentration (ΔSIC) for the seasons above, and **m**–**p** are the corresponding ensemble standard deviations in ΔSIC

Arctic warming projections display stark regional contrasts (Fig. 2) where sea ice retreat regions exhibit the greatest warming and possess the largest model disagreement. Figure 2 shows the annual and seasonal ensemble mean temperature and sea ice concentration (SIC) changes and the corresponding standard deviations across the ensemble. The Barents/Kara and Beaufort/Chukchi Seas regions (see map, Supplementary Figure 1) exhibit the largest projected warming and sea ice loss; wintertime temperature projections exceed +20 K in both regions. The central Arctic Ocean shows the largest seasonality of the temperature response, warming by 15–20 K in fall/winter and less than 5 K in spring/summer. During all seasons, the smallest temperature changes between 1–4 K occur in the sea ice-free ocean regions of the North Atlantic, Norwegian Sea, and Davis Strait due to small surface energy budget changes consistent with reductions in ocean heat transport^37^.

The spatial warming pattern varies significantly between models; some show larger temperature increases over the central Arctic Ocean and others show the greatest warming in regions of the largest sea ice loss. In general, the inter-model spread is greatest in the sea ice retreat regions (Fig. 2e–h), particularly the Barents/Kara and Beaufort/Chukchi Seas.

### Individual contributions to Arctic amplification

The presence of surface ice and the massive amounts of energy sequestered and released during water phase change indicates that the surface energy budget (SEB) is more relevant to the Arctic surface temperature than the top-of-atmosphere (TOA) energy budget. Previous work demonstrates that the surface and TOA perspectives can show opposite signs for the individual feedback contributions to warming, as Taylor et al.^38^ illustrated for clouds. Our SEB decomposition approach analyzes the energy flux changes and how they contribute to AA in each model (see Methods) by linearly decomposing the total surface temperature change into partial temperature contributions (PTCs). These terms include SAF, cloud radiative effect (CRE), changes in shortwave clear-sky radiation unrelated to SAF (SWCS), longwave clear-sky radiation (LWCS), ocean heat storage and transport (HSTOR), and surface turbulent fluxes (HFLUX, positive from atmosphere to ocean). In this decomposition, the LWCS term includes the effects of CO_2_, air temperature, and water vapor changes from both local and remote sources. We do not separate these effects as in previous work^15,18^ because biases due to the decomposition approach interfere with the assessment of inter-model differences. The HSTOR term represents the surface energy imbalance, including surface heat storage and ocean heat transport, computed as a residual (see Methods). Since the heat storage capacity of land is small compared to ocean, this term represents ocean heat content. Ocean heat transport, while potentially important to the inter-model spread, could not be assessed separately because few CMIP5 models archived the necessary output. This limits our ability to consider ocean heat storage and transport separately; however, previous work demonstrated that ocean heat storage in the mixed layer dominates changes in ocean heat transport in CMIP5 models over the 21^st^ century^15^; thus, we consider HSTOR changes to be from ocean heat storage.

Annual mean PTCs and formulae are listed in Table 1; PTCs are additive, each representing the individual feedback contributions to the total ensemble mean Arctic temperature change of 7.38 K by 2100 in RCP8.5. At least three times larger than any other feedback, the strongest annual surface warming contributions are from LWCS changes, 7.27 K. The SAF feedback exhibits the second strongest ensemble average annual warming contribution (1.82 K) and the cooling influence of HFLUX (−1.67 K) the third largest contributor.

**Table 1:** Annual mean partial temperature contributions for the Arctic domain (60°–90°N) from CMIP5 RCP8.5

Feedback	Partial temperature contribution formula	Annual mean partial temperature contribution (K)
Surface albedo feedback (SAF)	$\frac{{ - \left( {\Delta \alpha } \right)\left( {\overline {S_{{\mathrm{dn}}}} + \Delta S_{{\mathrm{dn}}}} \right)}}{{4\sigma T_s^3}}$	1.82 ± 0.77
Cloud radiative effect (CRE)	$\frac{{\left( {1 - \bar \alpha } \right)\Delta S_{{\mathrm{dn}},{\mathrm{cld}}} + \Delta F_{{\mathrm{dn}},{\mathrm{cld}}}}}{{4\sigma \bar T_s^3}}$	0.69 ± 0.88
Non-SAF shortwaves clear-sky feedback (SWCS)	$\frac{{(1 - \bar \alpha )\Delta S_{{\mathrm{dn}},{\mathrm{clr}}}}}{{4\sigma \bar T_s^3}}$	−0.43 ± 0.20
Longwave clear-sky feedbacks (LWCS)	$\frac{{\Delta F_{{\mathrm{dn}},{\mathrm{clr}}}}}{{4\sigma \bar T_s^3}}$	7.27 ± 1.40
Ocean heat storage (HSTOR)	$\frac{{ - \Delta Q}}{{4\sigma \bar T_s^3}}$	−0.30 ± 1.20
Surface turbulent fluxes (HFLUX)	$\frac{{ - \Delta (S + L)}}{{4\sigma \bar T_s^3}}$	−1.67 ± 0.86

**Fig. 3:**
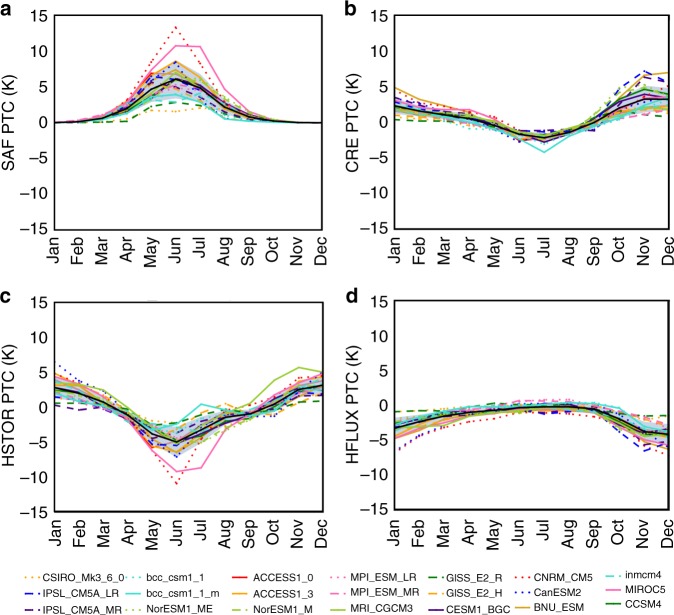
Seasonality of partial temperature contributions. Seasonal cycles of (**a**) surface albedo feedback, (**b**) cloud radiative effect, (**c**) ocean heat storage, and (**d**) surface turbulent flux partial temperature contributions averaged over the Arctic domain (60°–90° N). The gray shaded region denotes the ensemble mean (solid black line) +/− one standard deviation

The strong seasonality of the Arctic SEB renders the annual mean picture incomplete. Important factors influencing AA—HSTOR, CRE, and HFLUX—exhibit strong seasonal variations. Applying the decomposition methodology to monthly SEB changes enables the assessment of seasonal energy exchanges (Fig. 3).

**Fig. 4:**
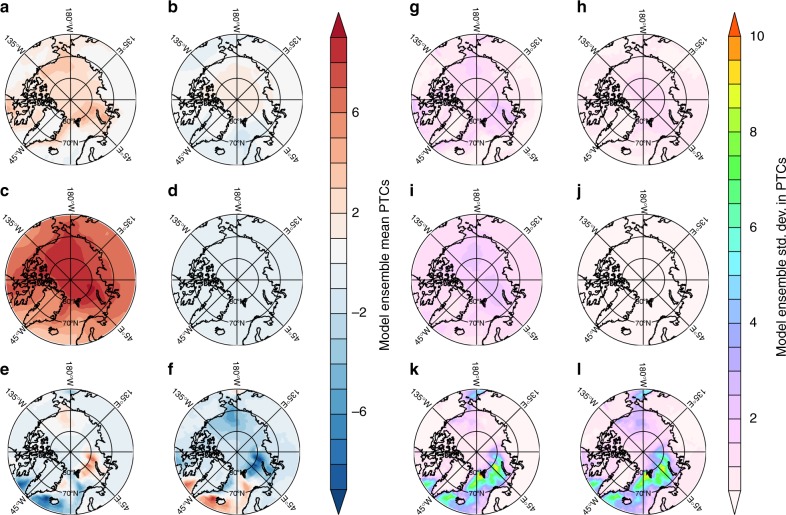
Spatial variablity of partial temperature contributions. Ensemble mean annually-averaged partial temperature contributions shown for (**a**) surface albedo feedback, (**b**) cloud radiative effect, (**c**) longwave clear-sky, (**d**) shortwave clear-sky, (**e**) ocean heat storage, and (**f**) surface turbulent flux. The corresponding inter-model standard deviation for each partial temperature contribution is shown for (**g**) surface albedo feedback, (**h**) cloud radiative effect, (**i**) longwave clear-sky, (**j**) shortwave clear-sky, (**k**) ocean heat storage, and (**l**) surface turbulent flux

The seasonality of the PTCs from CMIP5 remains unchanged from CMIP3 model analysis and is consistent with reanalysis^17,34,39^. Figure 3 shows a strong seasonality in SAF, CRE, HSTOR, and HFLUX. The SAF exhibits significant warming contributions peaking in June/July that mirror the increased HSTOR (Fig. 3a, c). While the SAF is partially offset by negative CRE PTCs (Fig. 3b), summer SAF increases the energy deposited into the ocean^14,40,41^. The negative HSTOR PTC (April–September) represents the accumulation of solar energy during spring and summer (more energy into the surface than out). Evidenced by the approximate equivalence of the combined SAF + CRE to HSTOR, changes in SAF and CRE primarily determine the summer HSTOR PTC. In fall/winter, HSTOR and HFLUX are approximately equivalent indicating that model HFLUX determines the fall/winter surface cooling rate change. From a process perspective, HFLUX is controlled by the air–sea temperature contrast, strongly influenced by both the presence of sea ice and atmospheric advection^16,42^. The negative fall/winter HFLUX PTCs in Fig. 4d are more than offset by positive fall/winter LWCS PTCs (not shown).

### Inter-model differences

The model spread between the contributions of individual SEB terms is represented by the inter-model standard deviation in Table 1. LWCS and HSTOR exhibit the largest annual mean, domain-averaged standard deviation, 1.4 K and 1.2 K, respectively. Considering percent differences however, the HSTOR inter-model spread is 400%. Clouds represent the second largest percentage spread at ~125%. Different from other feedbacks, the ±1 standard deviation bounds for these two feedbacks includes zero indicating that models disagree on the annual mean magnitude and sign of these contributions.

The strong seasonality of the PTCs renders the annual mean picture potentially misleading. Despite a modest annual mean inter-model spread (~40%), the SAF shows the widest inter-model range in any month, between +2 to +13 K in July (Fig. 3a). A strong correlation (*R* = −0.89) is found between annual mean albedo changes (peaking in summer) and AA (peaking in winter) even though the SAF seasonal cycle is out-of-phase with the maximum warming. The inter-model range in HSTOR (from −2 to −11 K) also maximizes in summer, aligning with the SAF inter-model spread. The inter-model spread in the CRE PTCs is the largest in fall (from +1 to +7 K) and smallest during summer. Models with the largest summer SAF do not exhibit the largest negative CRE PTC and therefore do not compensate for sea ice loss by simulating more reflective clouds. HFLUX exhibits its largest inter-model spread in winter (from −1 to −7 K) and smallest spread in summer (from −1 to +0.5 K). Seasonal cycles of the PTCs exhibit greater amplitude over ocean than land as in Laîné et al.^15^ (not shown), with HSTOR and HFLUX over the ocean having the largest inter-model range in winter of ~11 K between models. The SAF and HSTOR over the ocean show the largest inter-model range in summer of 18 K and 15 K, respectively.

### Spatial variability of process contributions

Regional differences are important because spatial patterns of sea ice loss affect Arctic climate variability and the warming response^43–45^. For instance, the pattern of sea ice loss modulates the position of regional baroclinic zones that are favored regions of cyclogenesis^46,47^. Moreover, Overland et al.^8^ suggest that the spatial pattern of warming and sea ice loss alters the mid-latitude circulation. Several recent studies indicate that local feedbacks in sea ice-retreat areas accelerate warming^16,48^. Moreover, future Arctic climate change is likely to occur in response to episodic energy fluxes, via surface turbulent fluxes and poleward heat transport^42,49,50^. Since these factors act on a regional level, it is important to assess model feedbacks and differences spatially.

Figure 4 shows the ensemble mean annually-averaged PTCs (**a**–**f**) and the corresponding spatial inter-model standard deviation for each PTC (**g**–**l**). The largest model differences occur in seasonal sea ice regions where small differences in sea ice extent correspond to large differences in HSTOR (Fig. 4k) and HFLUX (Fig. 4l). The most striking feature is the contrast between the spatial variability of PTCs and their associated inter-model spread for radiative and non-radiative feedbacks, revealing in almost every case that the spatial variation of non-radiative feedbacks (HSTOR and HFLUX) is stronger than for radiative feedbacks (SAF, CRE, SWCS, and LWCS). The spatial contours in Fig. 4 show that non-radiative feedbacks vary strongly across models, in sea ice-retreat regions the HSTOR standard deviation exceeds 10.0 in the annual mean, more than seven times the domain average, and also approaches 10.0 in the Barents/Kara Seas, more than five times the domain average. Thus, the inter-model spread in Arctic warming is strongly influenced by the non-radiative feedbacks (HSTOR and HFLUX) in sea ice-retreat regions.

**Fig. 5:**
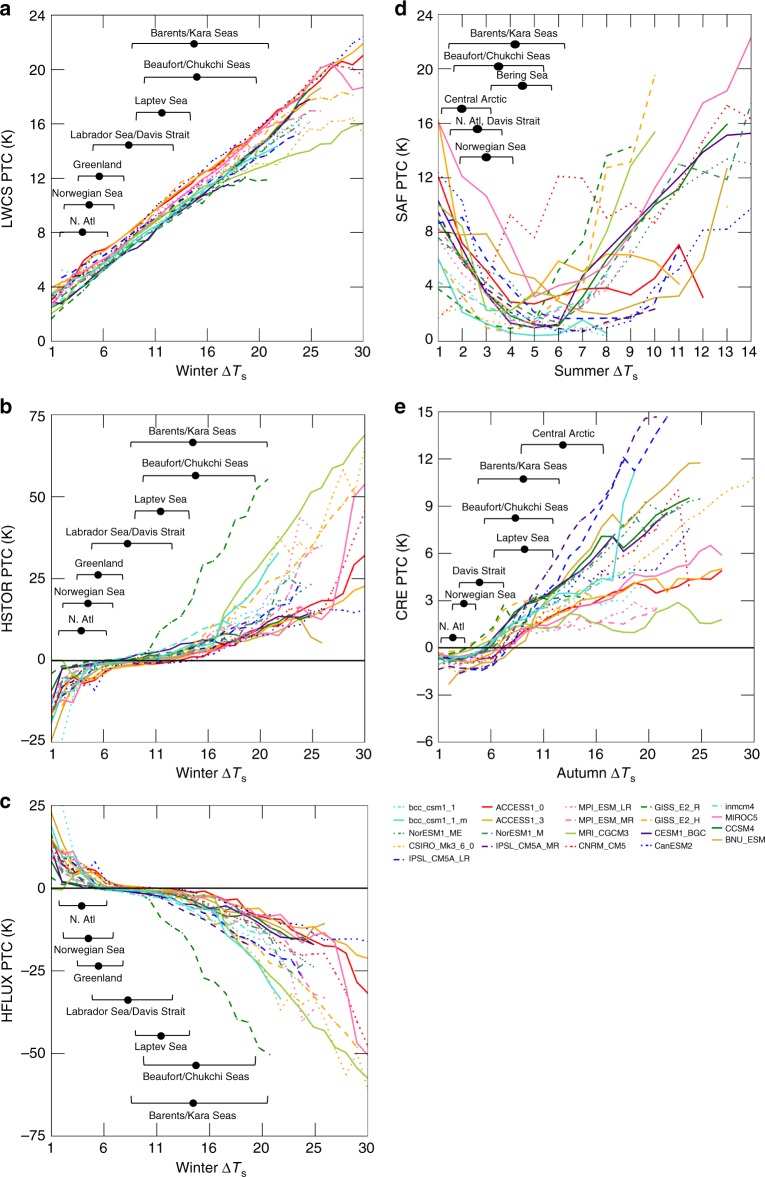
Relationship between regional warming pattern and individual partial temperature contributions. Select partial temperature contributions are plotted against projected temperature change by year 2100 using individual grid boxes for (**a**) winter longwave clear-sky, (**b**) winter ocean heat storage, (**c**) winter surface turbulent flux, (**d**) summer surface albedo feedback, and (**e**) autumn cloud radiative effect. The bracketed ranges show the ensemble mean temperature change (black filled circle) and the ±1 standard deviation for various Arctic regions; temperature changes for some individual models exceed this range

Sorting regional PTCs by the local warming amount (Fig. 5) indicates the most important terms driving the inter-model spread. Evident from Fig. 5, regions with the most warming exhibit the largest inter-model spread between the PTCs; the only exception being summer SAF (Fig. 5d). Finding the largest inter-model spread in regions that warm the most may not seem surprising; however, this result is not guaranteed. This result supports our conclusion that the mechanisms driving the inter-model spread in AA operate regionally.

Individual SEB terms contribute differently to the regional warming pattern. LWCS (Fig. 5a) and CRE (Fig. 5e) PTCs indicate a direct relationship with the regional warming pattern, where larger PTCs are found in regions of larger warming. Models overwhelmingly agree on the relationship between the regional warming and LWCS PTCs. The correspondence between regional characteristics of LWCS and warming indicates a direct local relationship exhibiting very little inter-model spread and supported by the small spatial variation in the LWCS PTC ensemble standard deviation (Fig. 4i). The CRE PTCs exhibit a larger inter-model spread and smaller PTCs than LWCS. Regions that warm the most show a positive year-round CRE PTC.

The SAF PTCs suggest a different relationship with regional warming. The summer SAF PTCs’ dependence on regional warming (Fig. 5d) resembles a ‘U-shape’—large PTCs in regions of small and large warming. Since regions that warm most in summer also warm most in fall/winter (Fig. 2), this suggests that the regional warming pattern itself is partially independent of the SAF, also supported by Kim et al.^34^.

HSTOR (Fig. 5b) and HFLUX (Fig. 5c) show monotonic relationships between PTCs and regional warming. HFLUX is negative (surface cooling) in regions that warm most and positive (surface warming) in regions that warm least, the opposite holds for HSTOR. Further, HFLUX and HSTOR PTCs are very close to zero in regions of modest warming, primarily land regions. This behavior indicates that changes in HFLUX control HSTOR and the regional surface heating/cooling rates. The largest inter-model spread in HSTOR and HFLUX PTCs are found in the regions of greatest warming (sea ice retreat regions) exhibiting an inter-model range exceeding 40 K. Overall, these results suggest that the regions that warm the most exhibit a strong summer SAF and fall/winter HFLUX and HSTOR PTCs.

### The process of Arctic amplification

Synthesis of our results and previous work paints a picture of AA whereby increased downwelling LW radiation (LWDN) dominates Arctic warming^17,18,29,51^. Observational evidence corroborates this model behavior demonstrating a significant contribution to recent Arctic warming and fall sea ice variability from LWDN^20,31,32,51,52^. Understanding the drivers of AA then reduces to quantifying the processes driving changes in LWDN.

**Fig. 6:**
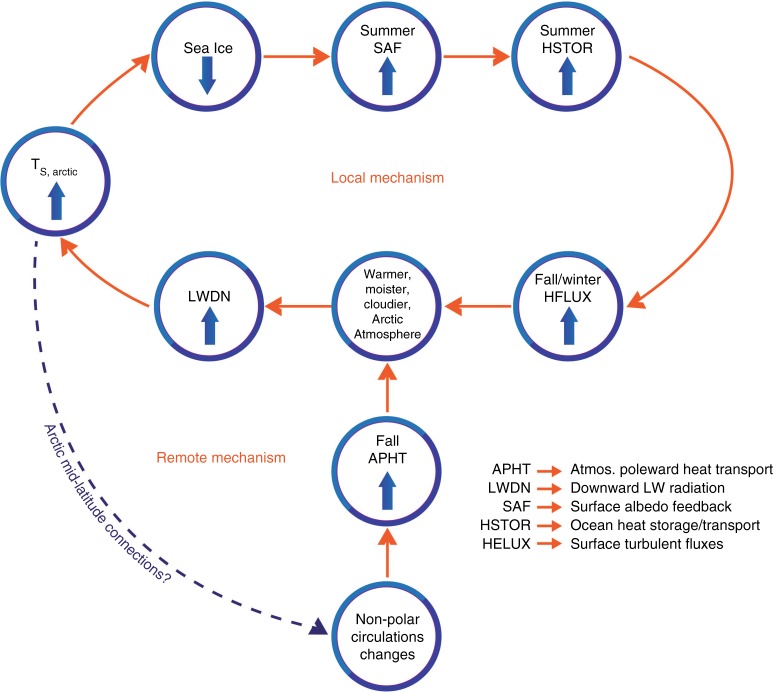
Local and remote Arctic amplification processes. The local and remote mechanisms represent two process loops that contribute to Arctic amplification. In the remote mechanism, changes in the non-polar circulation increase atmospheric poleward heat transport warming, moistening, and producing a cloudier Arctic, increasing longwave downward radiation. The local mechanism represents the combined surface albedo (radiative) and ice-insulation (non-radiative) feedbacks whereby a less sea ice covered Arctic stores more sunlight in the ocean in summer supporting increased surface turbulent fluxes in fall/winter, which warms, moistens, and produces cloudier conditions and increases longwave downward radiation. The common influence of both the local and remote mechanisms on longwave downward radiation links the two mechanisms facilitating constructive interference and makes them challenging to separate. A line connecting warmer Arctic temperatures with non-polar circulation changes suggests a potential feedback, however the dashed line is used to indicate the current lack of consensus on the magnitude and influence of Arctic temperature changes on the mid-latitude circulation

Figure 6 illustrates two primary process loops contributing to increased LWDN: remote and local mechanisms. In the remote mechanism, changes in the non-polar (tropical and mid-latitude) circulation increase atmospheric poleward heat transport (APHT) into the Arctic and warm, moisten, and produce a cloudier Arctic atmosphere, increasing LWDN. Proposed processes facilitating the increased APHT include mid-latitude circulation changes such as increased moisture intrusions^50^ and teleconnections with the tropical climate^53,54^. Atmospheric energy convergence from midlatitude moisture fluxes into the Arctic has increased LWDN and contributed to observed Arctic temperature trends between 1989 and 2009^48,51^.

The local mechanism represents the combined surface albedo (radiative) and ice-insulation (non-radiative) feedbacks whereby a warmer Arctic with less sea ice stores more energy in the ocean in summer via the SAF supporting increased HFLUX in fall/winter. The increased absorbed radiation is not immediately radiated away but stored and transferred from summer to fall/winter delaying fall sea ice freeze-up and providing an energy source to the atmosphere^11,55^. Increased HFLUX subsequently warms, moistens, and increases clouds, contributing to increased LWDN^16,34,39^. Increased LWDN and warmer Arctic temperatures in fall/winter promote thinner sea ice and further sea ice loss^48^, reinforcing the feedback loop by increasing the potential for ocean heat storage^55,56^.

Ocean mixed layer processes and heat transport modulate the local and remote mechanisms. The summer SAF warms the upper ocean making it less dense and more stably stratified. Heat entering the ocean is trapped near the surface, where it melts and thins sea ice [Graham et al. 2013]. Sea ice melt freshens the mixed layer and increases stratification encouraging the development of a near-surface temperature maximum^55,57,58^ —a layer of warm water below the shallow summer ocean mixed layer (OML). In fall, the OML cools, deepens, and transfers heat to the atmosphere via HFLUX and longwave radiation^59^. More ice-free ocean supports greater upper ocean mixing by atmospheric winds, which can entrain warm water from the near-surface temperature maximum layer and delay fall sea ice freeze-up^55,58^. However, this process is likely poorly represented across CMIP5 models due to insufficient upper ocean vertical mixing^60^.

Ocean heat transport into the Arctic influences temperature and sea ice, passes energy to the atmosphere via HFLUX, and modifies the Arctic circulation. Reconstructions of long-term records^27^ and model output point to ocean heat transport as a potential source of AA and inter-model spread^6,61^. Oceanic heat transport into the Barents Sea influences Arctic climate variability and the North Atlantic Oscillation^62,63^ by reducing sea ice, enhancing HFLUX, and lowering the local atmospheric pressure. However, ocean heat transport in CMIP5 models contributes little to projected warming over 21^st^ century^15^. These interactions suggest that changes in the position and intensity of the jet stream and frequency of synoptic cyclones can modulate the local and remote mechanisms by influencing sea ice, HFLUX, and the OML, representing a process link between the two mechanisms.

The fact that the remote and local mechanisms contribute to AA via increased LWDN (Fig. 6) also links these two mechanisms^29,64^. It is clear that the remote forcing mechanism can accelerate the local mechanism by increasing LWDN and influencing sea ice^52^. It is an open question if changes in the Arctic drive changes in the mid-latitude circulation (dashed line in Fig. 6)^65^. Zappa et al.^66^ suggest that sea ice loss in CMIP5 models influences the position of the midlatitude westerly jet, promoting an equatorward shift and consistent with previous work^67,68^. These links suggest the potential for constructive and destructive interference between these mechanisms, however it is unclear how this interference influences the inter-model spread and cannot be assessed from the current methodology. We hypothesize that quantifying the strength of interactions between the local and remote mechanisms in observations is key to determining if the largest projected AA is likely. This is an important area for future work.

### Drivers of inter-model spread

The largest inter-model differences in the warming response (Fig. 2) and feedback contributions (Fig. 4) are found in sea ice retreat regions. These regions exhibit the largest summer SAF and fall/winter HFLUX/HSTOR PTCs, as well as the largest LWCS increases (Fig. 5). The HFLUX and HSTOR PTCs also show the largest inter-model differences in sea ice retreat regions. These factors suggest that the local mechanism drives the inter-model spread.

**Fig. 7:**
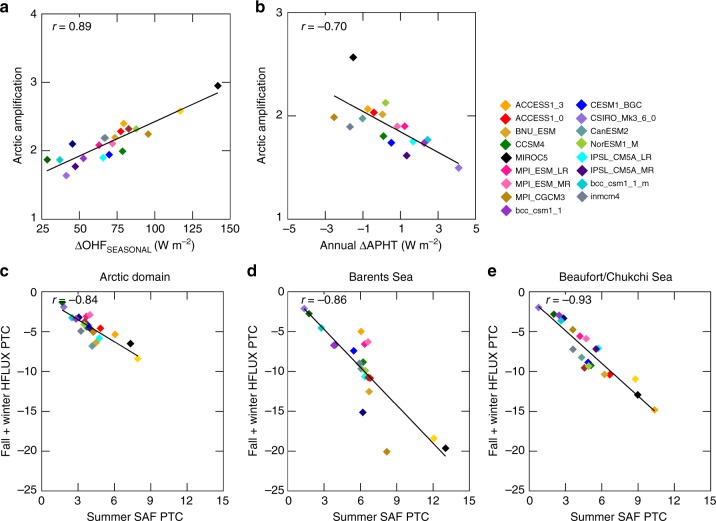
Energy transfer and Arctic amplification. **a** Correlation between the changes in seasonal ocean heat flux amplitude (ΔOHF_seasonal_) and Arctic amplification. **b** Correlation between annual change in atmospheric poleward heat transport and correlation between model summer surface albedo feedback partial temperature contribution and fall/winter surface turbulent flux partial temperature contribution for (**c**) Arctic domain, (**d**) Barents Sea, and (**e**) Beaufort/Chukchi Sea

The local mechanism links the SAF, HSTOR, and HFLUX terms through seasonal energy transfer. To test our hypothesis that differences in the local mechanism explain the AA inter-model spread, we identify a metric based upon the amplitude of seasonal energy exchanges—the seasonal ocean heat flux (OHF_SEASONAL_). OHF_SEASONAL_ is defined as the difference between the month of minimum and maximum HSTOR and ΔOHF_SEASONAL_ represents its change by 2100. Figure 7a shows a statistically significant relationship between the ΔOHF_SEASONAL_ and AA (*r* = 0.89), supporting our hypothesis. Using ΔOHF_SEASONAL_ as an observational constraint on projected AA may lack practical relevance if the signal emergence does not occur before warming. Conversely, the remote forcing mechanism, represented by ΔAPHT (see Methods), anticorrelates with AA suggesting that it dampens the inter-model spread (Fig. 7b). Thus, the seasonal exchanges of energy related to the local feedback mechanism widen the inter-model spread in AA.

Models increase ΔOHF_SEASONAL_ through a stronger summer SAF and a larger fall/winter HFLUX (Fig. 7c). This seasonal energy transfer is amplified in sea ice retreat regions, such as the Barents/Kara and Beaufort/Chukchi Seas regions (Fig. 7d, e) and does not operate in ice-free ocean areas (not shown). Therefore, models accomplish a stronger seasonal transfer of energy through a larger summer SAF, storing heat in the ocean and enhancing fall/winter HFLUX in sea ice retreat regions.

**Fig. 8:**
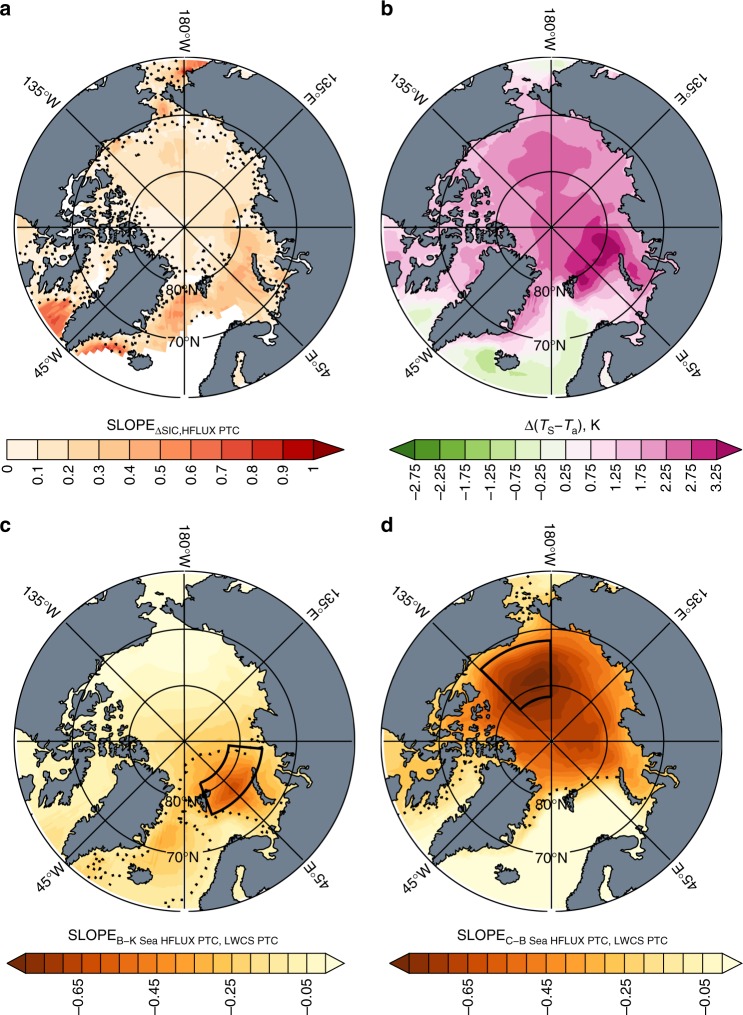
Dispersal of energy from sea ice retreat regions to the broader Arctic. **a** Regression slope between annual mean change in sea ice concentration and fall surface turbulent fluxes. **b** Ensemble mean change in the air–sea temperature (*T*_s_ − *T*_a_) gradient. **c** Regression slope between the fall surface turbulent flux change partial temperature contribution in the Barents/Kara Seas (boxed region) and the fall longwave clear-sky partial temperature contribution across the ensemble and **d** as is **c** for the Beaufort/Chukchi Seas. Regions where the regression slope is significant at the 90% level are bounded by the dotted line. The statistically significant regression slopes suggest that models with larger changes in surface turbulent fluxes in the Barents/Kara and Beaufort/Chukchi Seas also produce larger increases in the downward longwave clear-sky flux across the broader Arctic

Figure 8 illustrates the series of relationships that contribute to the inter-model spread due to the local mechanism in sea ice retreat regions. First, the SAF and increased summer heat storage cannot directly increase HFLUX, rather increased summer heat storage supports a delayed fall sea ice freeze-up and a positive fall/winter air–sea temperature gradient (*T*_s_ − *T*_a_). Figure 8a illustrates that models with less fall/winter sea ice produce larger HFLUX. Increased HFLUX is supported by stronger air–sea temperature gradients (Fig. 8b) that are maintained by the atmospheric circulation through advection.

Inter-model differences in sea ice retreat regions have non-local effects through the atmospheric circulation. Increased HFLUX warms, moistens, and produces clouds locally, increasing LWDN, deepening the OML, and contributing to a delayed fall sea ice freeze-up. The influence of local HFLUX changes on the Arctic-wide LWCS requires the atmospheric circulation to disperse this energy. Figure 8c, d shows a statistically significant linear regression slope between model fall/winter HFLUX PTC in the Barents/Kara and Beaufort/Chukchi Seas and the spatial pattern of ΔLWCS; models with a larger magnitude fall/winter HFLUX PTC in sea ice retreat regions generate a larger Arctic-wide LWCS PTC. Comparing Fig. 8c, d indicates a larger Arctic-wide LWCS PTC due to ΔHFLUX in the Beaufort-Chukchi Seas, suggesting that the atmospheric circulation is more sensitive to a perturbation in this region. The anti-correlation between ΔOHF_SEASONAL_ and ΔAPHT (Fig. 7) suggests that the inter-model differences in how this energy is dispersed are not from the large-scale circulation, but due to the local circulation. We infer that local atmospheric circulation differences contribute to the inter-model spread in AA by influencing how energy drawn from the surface in sea ice retreat regions is dispersed. Burt et al.^16^ hypothesize a possible mechanism whereby reduced fall/winter sea ice induces a thermal contrast between the Arctic Ocean and the colder Arctic continents driving a circulation response, termed a shallow winter monsoon, promoting stronger HFLUX.

**Fig. 9:**
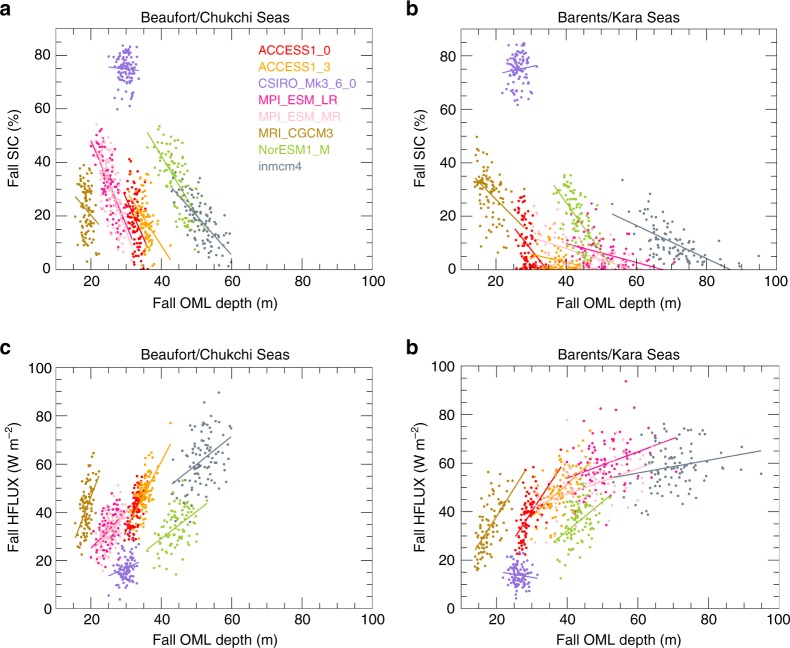
Implications of changes in ocean mixed layer depth. Relationships between ocean mixed layer depth, sea ice concentration, and surface turbulent flux in sea ice retreat regions, showing (**a**) Beaufort/Chukchi Seas fall mixed layer depth and fall sea ice concentration, (**b**) Barents/Kara Seas fall mixed layer depth and fall sea ice concentration, (**c**) Beaufort/Chukchi Seas fall mixed layer depth and fall surface turbulent flux, and (**d**) Barents/Kara Sea fall mixed layer depth and fall surface turbulent flux. A linear regression fit is drawn for each model (solid lines). The results indicate significant inter-model differences in the present-day and future, changes in ocean mixed layer depth as well as the relationship with sea ice concentration and surface turbulent fluxes

OML depth modulates the seasonal energy transfer and can impact the inter-model spread in AA. Eight CMIP5 models that archived OML depth show very large inter-model differences in the relationships between OML depth, SIC, and HFLUX in Barents/Kara and Beaufort/Chukchi Seas regions. A deeper OML contains a higher heat capacity supporting larger HFLUX and less sea ice (Fig. 9). Moreover, the average OML depth in the Barents/Kara Seas region ranges from 13 to 95 m in fall. Motivated by this inter-model spread and the relationship in Fig. 7, we hypothesized that the OML depth influences the inter-model spread in AA by modulating the seasonal energy transfer. However, we found no correlation between the mean state OML depth or its change with AA. The lack of correlation may indicate a bias in the model representation of OML dynamics, such as the known bias in upper ocean vertical mixing^60^. Despite the lack of correlation, this range of OML depth significantly influences the ability of the ocean to store energy and modulate surface turbulent fluxes, sea ice variability, and the atmospheric circulation. Therefore, an improved understanding of the causes and consequences of the inter-model spread in OML depth and its relationship with sea ice and surface turbulent fluxes is needed.

## Discussion

Our results indicate that the local AA mechanism—the combined sea ice albedo and ice insulation feedbacks—significantly contributes to the inter-model spread in AA. The remote AA mechanism is shown to dampen the inter-model spread.

We conclude that models that transfer more energy from summer to fall produce a larger AA. This seasonal energy transfer is primarily accomplished by increased absorption and storage of solar insolation in summer and increased surface turbulent fluxes in fall/winter in sea ice retreat regions. Models simulating greater reductions in summer/fall sea ice and larger fall/winter surface turbulent fluxes produce more AA. Our results suggest that the local Arctic circulation and its response contribute to the inter-model spread in AA by dispersing the energy drawn from the surface in sea ice retreat regions Arctic-wide and reinforcing the local air–sea temperature gradients. Models that more widely disperse the energy drawn from the surface in sea ice retreat regions warm more. We found significant inter-model differences in ocean mixed layer depths and its relationships with sea ice concentration and surface turbulent fluxes that modulate seasonal energy transfer, yet do not correspond to the inter-model spread in AA. This lack of correlation suggests a bias in the model representation of the ocean mixed layer with the potential to significantly impact projected AA.

This explanation contains familiar arguments for the processes that drive the AA inter-model spread; however, the regional picture presented should not be overlooked. The local mechanism does not occur throughout the Arctic but is focused in regions of sea ice retreat. Neglecting the regional variation in AA mechanisms paints an incomplete picture, as the inter-model spread in warming possesses a regional structure. Only after considering the regional perspective does the importance of the local atmospheric circulation to AA become clear. Our results suggest that an atmospheric circulation change local to the Arctic may be an important factor in the inter-model spread. While much of the scientific literature attempts to explain inter-model differences in AA using atmospheric-only mechanisms, we contend that a complete theory of AA and its inter-model spread must consider the atmosphere-ocean-sea ice system. Our results indicate that the principle mechanisms driving the inter-model spread in AA operate on regional not Arctic-wide scales and that reductions in the inter-model spread in projected Arctic warming require an improved process representation of atmosphere-ocean-sea ice interactions in sea ice retreat regions.

## Methods

### Surface energy budget decomposition

Data analyzed during this study comes from 21 CMIP5 models, listed in Supplementary Table 1, which archived the required data to perform the surface energy budget decomposition^10^. All data are available from the Earth System Grid Federation Peer-to-Peer enterprise system at https://esgf-node.llnl.gov/projects/esgf-llnl/. The model simulations were forced with RCP8.5, a high-emission scenario from 2006 to 2100. Present (future) climatology was determined from the average of the first (last) 20 years of the simulation. Using available data, surface energy budget can be computed as

1 $$Q = \left( {1 - \alpha } \right)S_{{\mathrm{dn}}} + F_{{\mathrm{dn}}} - \varepsilon \sigma T_s^4 - \left( {S + L} \right),$$

where *Q* represents the storage of heat for all surface types as well as oceanic transport, *α* is the surface albedo defined by the ratio of upward to downward shortwave clear-sky fluxes, *S*_dn_ is incident solar radiation, *F*_dn_ is the downwelling longwave radiation, *εσT*_s_^4^ is the longwave emission from the surface at temperature *T*_s_ (where the emissivity, *ε*, is assumed to be equal to 1), and (*S* *+* *L*) are the sensible and latent heat fluxes (defined as positive upward). All variables are available from the CMIP5 data portal except for *Q*, which is obtained as a residual. After solving for *T*, a perturbation form of (1) yields

2 $$4\sigma T_{\mathrm{s}}^3\Delta T = \Delta \left[ {\left( {1 - \alpha } \right)S_{{\mathrm{dn}}}} \right] + \Delta F_{{\mathrm{dn}}} - \Delta Q - \Delta \left( {S + L} \right),$$

where Δ-values represent the change in a variable between present-day and future.

Following Lu and Cai^17^, the change in the cloud radiative effect (CRE) is calculated as

3 $$\Delta{\mathrm{ CRE}} = \left( {1 - \overline{ \alpha} } \right)\Delta S_{{\mathrm{dn}},{\mathrm{cld}}} + \Delta F_{{\mathrm{dn}},{\mathrm{cld}}},$$

where $\Delta S_{{\mathrm{dn}},{\mathrm{cld}}}\,{\mathrm{and}}\,\Delta F_{{\mathrm{dn}},{\mathrm{cld}}}$ are computed as all-sky minus clear-sky radiative fluxes and ${{\bar \alpha }}$ represents mean state surface albedo. This formulation of ΔCRE controls for the influence of surface albedo on CRE. Substitution of (3) into (2) and dividing by 4*σT*_*s*_^3^ yields

4 $${ \Delta T = \frac{{ - \left( {\Delta \alpha } \right)\left( {\overline {S_{{\mathrm{dn}}}} + \Delta S_{{\mathrm{dn}}}} \right) + \Delta {{CRE}} + \left( {1 - \overline{\alpha} } \right)\Delta S_{{\mathrm{dn}},{\mathrm{clr}}} + \Delta F_{{\mathrm{dn}},{\mathrm{clr}}} - \Delta Q - \Delta \left( {S + L} \right)}}{{4\sigma T_s^3}}}.$$

Each term on the right-hand side of (4) represents a partial temperature contribution for SAF, CRE, changes in shortwave clear-sky radiation unrelated to SAF, changes in longwave clear-sky radiation, changes in heat storage, and changes in surface turbulent fluxes, respectively (Table 1). Each PTC represents the temperature contribution of the corresponding feedback to the total Arctic temperature change by 2100.

### Calculation of atmospheric poleward heat transport (APHT)

The annual mean net radiation balance for latitudes poleward of ~40° is negative, meaning it emits more energy than it receives, requiring poleward heat transport. The energy balance of the Arctic domain can be written as in (5)

5 $$\frac{{\partial E}}{{\partial t}} = R_{{\mathrm{TOA}}} - F_{{\mathrm{ao}}}$$

where $\frac{{\partial E}}{{\partial t}}$ is the time rate of change in energy content of the Arctic, *R*_TOA_ is the net incoming radiation at TOA, and *F*_ao_ is the horizontal flux divergence for atmosphere and ocean (poleward heat transport term). Averaging over timescales greater than one year makes the energy storage term $\frac{{\partial E}}{{\partial t}}$ negligible and then the implied poleward heat transport (PHT) is calculated by requiring a balance between *R*_TOA_ and *F*_ao_. *R*_TOA_ is calculated using CMIP5 model outputs at TOA for downwelling shortwave radiation (*S*_TOA,dn_), upwelling shortwave radiation (*S*_TOA,up_), and upwelling longwave radiation (*F*_TOA,up_). The equation for the combined atmospheric and oceanic poleward heat transport then becomes

6 $$F_{{\mathrm{ao}}} = R_{{\mathrm{TOA}}} = - \left( {S_{{\mathrm{TOA}},{\mathrm{dn}}} - S_{{\mathrm{TOA}},{\mathrm{up}}} - F_{{\mathrm{TOA}},{\mathrm{up}}}} \right).$$

The atmospheric component of poleward heat transport (*APHT*) is determined by taking the difference between *R*_TOA_ from the surface energy imbalance. After averaging over timescales greater than one year and neglecting $\frac{{\partial E}}{{\partial t}}$, Eq. (5) becomes

7 $$APHT = - \left( {R_{{\mathrm{TOA}}} - R_{{\mathrm{SFC}}}} \right)$$

where

8 $$R_{{\mathrm{SFC}}} = F_{{\mathrm{net}}} + S_{{\mathrm{net}}} - \left( {S + L} \right).$$

### Code availability

Computer code used for the analysis was written in IDL and is available from the authors upon request.

## Electronic supplementary material

Peer Review File

Supplementary Information

## Data Availability

The CMIP5 model data analyzed and support the finding of this study are deposited in the Earth System Grid Federation Peer-to-Peer enterprise system and available at https://esgf-node.llnl.gov/projects/esgf-llnl/.
