# Supplementary material for: Seasonal energy exchange in sea ice retreat regions contributes to differences in projected Arctic warming
Source: Nat Commun. 2018 Nov 27;9:5017. doi: 10.1038/s41467-018-07061-9 (PMC6258731; doi:10.1038/s41467-018-07061-9)
Supplement: Supplementary file 2 — Supplementary Information [file 41467_2018_7061_MOESM2_ESM.pdf]

Reviewer #1 (Remarks to the Author):

The manuscript presents a decomposition of Arctic warming and its inter-model spread in CMIP5 models into contributions from longwave/shortwave clear-sky and all-sky radiative fluxes and turbulent surface fluxes. The authors conclude that turbulent fluxes and thus atmosphere-ocean interaction in the Barents-Kara seas play a crucial and previously overlooked role in controlling the inter-model spread in Arctic amplification. Understanding inter-model spread in Arctic amplification is a relevant current research question, which can be advanced by the manuscript. I therefore recommend it for publication after the following major issues have been addressed:

1. The relationship between Delta OHF and AA appears dominated by the outlier Miroc 5. How robust is that relationship if this model is excluded?

2. More care is needed in the mechanistic interpretation of the results. I wasn't quite convinced that Fig. 6 and the discussion thereof demonstrate that spread in Delta OHF causes spread in AA (and not the other way around). The conclusion that atmosphere-ocean fluxes and ocean mixed-layer processes must be improved in models is not quite supported by the presented data and arguments.

3. The authors argue that Delta OHF could be an observational constraint for AA – however, for that to be of practical relevance, it would have to be demonstrated that the signal in Delta OHF emerges earlier than that in Arctic-wide temperature change.

Minor issues:

1. title: I would suggest referring to the inter-model spread here.

2. ll. 55 ff. Maybe cite earlier work on the albedo effect, too (Budyko, Arrhenius).

3. l. 79. I believe there is a plot on the seasonal cycle in Pithan and Mauritsen 2014.

4. l. 103 ff. Should it be considered that zonal means near the pole are an average over a much smaller area than at lower latitudes?

5. l. 149 Graversen

6. table 1: I suggest naming the institute rather than the country, and maybe referring to the atmospheric model in use, too (to make model similarities more transparent).

Reviewer #2 (Remarks to the Author):

The paper is attempting to assess contributions of various processes to polar amplification based on a host of CMIP5 models. An approach somewhat similar to one suggested my Ming Cai in his 2005 paper is utilized. Analysis suggests that the 'hotspot' that contributes the most to the warming in the Arctic is roughly collocated with the Barents-Kara seas. This is not a new result, which is one of the reasons why the paper should be rejected. The analysis raises certain questions as well (see below). The paper is difficult to read and follow: too many acronyms, the text flow is not smooth and sometimes it is hard to see the logic behind authors' arguments. I suggest rejecting the paper. Below are my more detailed comments.

How does the utilized 'feedback analysis' relate to the classical approach described in Peixoto Oort "Physics of Climate"? All I can see is the analysis of the surface heat budget. This is actually an extremely poor approximation to M.Cai's approach described in his 2005 paper. No dynamics of any kind is included. Non-locality of any kind can not be described IN PRINCIPLE using a local surface heat budget equation.

What the authors did is the following. They calculated all the surface fluxes in the 'present day' and future climates and claim that changes in the fluxes can serve to explain various feedbacks. The fundamental question here is whether the fluxes can be considered as forcings affecting the climate or they are a part of the climate system responding to changes. Do they form independently of the state of the climate? I would argue they do not. Therefore they cannot be separated ino a special category and/or be considered as forcings independent of the response (or the change of the system). The whole approach needs to be justified on a much more solid basis.

What do the authors mean by regional in the title? If the main 'hotspot' is in the Barents-Kara seas, how does that signal spread all over in the entire Arctic? How does the ocean contribute to forming the 'hotspot'? How local is this contribution from the ocean? Does the North Atlantic play a role and if it does, how local is the signal in the Barents-Kara seas?

What are those 'interactions' in the title? It is not clear what the authors mean.

Some deeper background research of the topic is recommended. A number of papers (even before any of M.Cai's papers) that actually describe and explain polar amplification mechanisms should have been included in the introduction. The Graversen et al (2008) Nature paper cannot be used as the one that introduced the arctic (or polar) amplification. Besides, it is also well known that the

analysis described in that paper is not quite free of flaws. Note that R.G.Graversen's name is misspelled (line 65).

Arrhenius S (1896) On the influence of carbonic acid in the air upon the temperature of the ground. Phil Mag 41:237–276

Flannery BP (1984) Energy-balance models incorporating transport of thermal and latent energy. J Atm Sci 41:414–421

Schneider EK, Lindzen RS, Kirtman BP (1997) A tropical influence on global climate. J Atm Sci 54:1349–1358

Rodgers KB, Lohmann G, Lorenz S, Schneider R, Henderson GM (2003) A tropical mechanism for Northern Hemisphere deglaciation. Geochem Geophys Geosyst 4(5):1046. DOI:10.1029/2003GC000508

Alexeev VA, Langen PL, Bates JR (2005) Polar amplification on surface warming on an aquaplanet in ‘‘ghost forcing’’ experiments without sea ice feedbacks. Clim Dyn.

doi:10.1007/s00382-005-0018-3

Reviewer #3 (Remarks to the Author):

Review of the manuscript entitled “Projected Arctic warming controlled by regional atmosphere-ocean interactions” by R.C. Boeke and P.C. Taylor

Research aimed at an assessment of relative contribution of individual feedbacks to Arctic Amplification (AA) is important and timely. However, I’m concerned with the applicability of CMIP5 model simulations for the pan-Arctic region to advance it. Specifically, given the relatively coarse spatial and temporal model resolution of CMIP5 model components over the Arctic, I remain skeptical about their usefulness to assess the relative impact of processes and feedbacks on the

magnitude of AA. Especially, when the primary diagnostics for those contributions and impacts is the inter-model spread in simulated AA, as elaborated below.

Moreover, I find it troubling that the term in the surface energy budget, the ocean heat storage and transport, critical to the conclusions of this paper is not calculated directly from model output but estimated as a residual from the surface energy imbalance. This makes it impossible to understand and assess the relative importance of specific processes and feedbacks controlling the ocean heat storage and transport and their contribution to AA. While the authors provide the justification for their approach, i.e. the lack of proper CMIP5 model output, the problem remains that they conclude on the importance of the term, which is not directly calculated from models, nor can be directly evaluated for its contribution to the model spread in simulating AA.

Also, I find the concept of the Arctic Amplification Index and results shown in Fig.5 overly complicated and hard to interpret physically. Given that these results are critical to the main conclusions of this paper, I believe they could be either better explained or presented by alternative means, easier for physical interpretation. In particular, the inset maps of the annual and seasonal standard deviation among models for each AAI in Fig.5 show largest magnitudes of HSTOR and HFLUX in the Greenland Sea throughout the year and also in the Labrador Sea during winter (JF), which is not discussed in the text and different from the spatial pattern of the inter-model standard deviation of projected surface temperature change shown in Fig.3e-h

Given the importance of changes in the sea ice cover to AA, I argue it’s critical to show how CMIP5 models represent it (preferably using ice thickness distribution) for the first and last 20 years of simulation. One key reason for that is to demonstrate model skill in representing the present time spatial distribution of sea ice and its change by 2100, which will not only affect AA through local feedbacks but will also reflect on model biases and spread. For example, many if not all CMIP5 models, likely experience similar biases in representing northward oceanic heat transport from the Pacific through (narrow and shallow) Bering Strait, which should translate onto their simulation of sea ice distribution in the western Arctic but not be necessarily revealed in the model spread.

In summary, I find this manuscript addressing an important scientific and societal problem and worth publication in Nature Communications, subject to major revision in order to address the main concerns above. An extended discussion of the limitations of CMIP5 models in representing processes and feedbacks of relevance to AA, including those controlling the two terms, HSTOR and HFLUX, would be a critical part of this revision.

Detailed comments:

- L.27-32: How well the ocean heat storage and mixed layer processes as well as surface turbulent fluxes and air-sea interactions are represented in the CMIP5 climate models?

- L.34-35: what evidence exists or can be provided to support the 2nd conclusion?

- L.109-112 and Fig. 3: explain and justify the criteria for defining seasons as in Fig. 3, specifically the sunlit (MAMJJAS) season?

- L.112-118: explain what observational data have been used to constrain the model spatial patterns and magnitudes f AA against reality?

- L.116-121: clarify the contradicting statements about the warming in the Norwegian Sea in these two sentences;

- L.120-121: clarify/define what 'heat transport from lower latitudes' is referred to here and present or discuss model results on those transports;

- L.126: discuss measures / metrics to support the statement about the SEB being more relevant to the state of the Arctic system, compared to the TOA energy budget;

- L.132: please consider changing the name of HSTOR from "surface" to "ocean" heat storage and transport, since the surface is just an interface so it can't store or transport properties.

Reviewer #4 (Remarks to the Author):

Review of "Projected Arctic warming controlled by regional atmosphere-ocean interactions" by Boeke and Taylor.

General

This is nice and informative paper on the reasons of the intermodel differences in Arctic Warming (AW). This, they show, can be attributed to regional surface heat fluxes. I believe this is a new(ish) insight (see comments below), and the paper is very well written, and I have very little comments on their analyses of the models, which seem sound and complete. That being said, I have a couple of other specific comments, which should be addressed before the paper would be, in my view, suitable for publication.

Specifics

1. Roughly the first half of the paper (Figs. 1-3) reads as an introduction, or even a repetition of results long known. In fact, on line 155 they even state that the results presented here, based on CMIP5 models, basically replicate those of Lu/Cai, 2009, which were based on CMIP3 models. While reading this first half, I got the impressing that this part can be significantly shortened with some proper referencing. Even though the first half reads very well, it is also evoking thoughts as “when does the new stuff start”, at least with me. Hence, I strongly suggest to shorten this part a great deal.

2. While I understand that the paper focuses on intermodel differences, it would still be nice to have the model-mean somewhere as a line or value in the various figures/tables, if only for readers to see how far a particular model if ‘off’ the model mean.

3. In Fig. 7, one of the 3 maps in panel a seems to be missing.

4. One critically missing issue relates to sea ice. Obviously, the huge changes in the BK seas in surface heat flux and storage are related to sea ice retreat (and thinning), and the intermodel differences in the surface heat fluxes are therefore related to those in sea ice retreat. The authors are well aware of this, I would assume, and it is indeed mentioned, but only, seemingly, as a side note. I believe sea ice retreat is crucial to the processes at hand, and should be discussed (and shown!!) much more prominently. One would think that models with the greatest changes in surface heat flux would also exhibit the largest sea ice retreat (and thinning), related to greater warming…

5. Related to 4… The authors state that changes in surface heat fluxes (in the BK seas) control warming, but how are they so sure of the causality? Models that for some reason exhibit greater AW also have stronger sea ice retreat, and thus bigger changes in surface heat fluxes. So the changes in the heat fluxes may equally well be the result of AW, not the cause (or both). More analyses is required to demonstrate the hypothesized causality, perhaps by analysing leads/lags.

6. The authors seem to have overlooked recent research that relates variations in surface heat fluxes in the BK region to Arctic Decadal Variability (ADV), see e.g. http://onlinelibrary.wiley.com/doi/10.1002/2016JD026058/full. The finding that the surface fluxes are key in ADV may relate to the issue presented here in the sense that the atmosphere-ocean interactions in the BK area are crucially important for Arctic climate, so for both AW and variability. This would not surprise me at all. I think this link (equivalent processes being crucial for AW and variability) is worth looking into.

Reviewer #1 (Remarks to the Author):

The manuscript presents a decomposition of Arctic warming and its inter-model spread in CMIP5 models into contributions from longwave/shortwave clear-sky and all-sky radiative fluxes and turbulent surface fluxes. The authors conclude that turbulent fluxes and thus atmosphere-ocean interaction in the Barents-Kara seas play a crucial and previously overlooked role in controlling the inter-model spread in Arctic amplification. Understanding inter-model spread in Arctic amplification is a relevant current research question, which can be advanced by the manuscript. I therefore recommend it for publication after the following major issues have been addressed:

1. The relationship between Delta OHF and AA appears dominated by the outlier Miroc 5. How robust is that relationship if this model is excluded?

In our revisions of this paper, we have added additional models. Figure 7 shows that this correlation discussed in the originally submitted paper still holds after adding additional models. Additionally, the strong, statistically significant correlation remains after removing the MIROC5 model from the correlations.

2. More care is needed in the mechanistic interpretation of the results. I wasn't quite convinced that Fig. 6 and the discussion thereof demonstrate that spread in Delta OHF causes spread in AA (and not the other way around). The conclusion that atmosphere-ocean fluxes and ocean mixed-layer processes must be improved in models is not quite supported by the presented data and arguments.

We have significantly revised this discussion and have included additional figures and analysis (Figs. 6-9). Our discussion includes more details as well as more references. Our conclusion now includes the importance of seasonal energy exchanges in sea ice retreat regions and the dispersal of this energy by the local atmospheric circulation as the key processes driving the inter-model spread. The additionally analysis that was suggested found no correlation between changes in ocean mixed layer depth and AA, however Fig. 9 does show very large inter-model differences in the relationships between the ocean mixed layer depth, surface turbulent heat fluxes, and fall sea ice.

Our argument follows that models that increase the seasonal amplitude of ocean heat storage (more storage in summer, more release in fall/winter) produce more Arctic amplification (Fig. 7). We now explicitly show that models accomplish this increased seasonal amplitude through a combination of the summer surface albedo feedback and fall/winter HFLUX (Fig. 7). This relationship is evident Arctic wide, but strongest in sea ice retreat regions (Barents-Kara, and Beaufort-Chukchi Seas). Moreover, enhanced ocean heat storage in summer contributes to delayed fall sea ice freeze-up and reduced fall/winter sea ice concentration (Fig. 9). Increased surface turbulent fluxes are driven by an increased air-sea temperature gradient (Fig. 8) exhibiting the strongest ensemble mean changes in sea ice retreat regions. To this point, the processes discussed are local actors. Influencing Arctic wide warming requires that energy drawn from the ocean in sea ice retreat regions be dispersed throughout the Arctic. Figure 8 shows the across model correlation between changes in surface turbulent fluxes in the Barents-

Kara Seas and Beaufort/Chukchi Seas regions and local changes in LW clear sky downwelling PTC, suggesting that enhanced surface turbulent fluxes in sea ice retreat regions influences a wider region of the Arctic.

How does the atmosphere redistribute this energy? Figure 7 shows an anti-correlation between the atmospheric poleward heat transport with Arctic Amplification indicating that it is not responsible for the redistribution of energy that enhances warming. Therefore, we hypothesize that a local atmospheric circulation response, one that does not change poleward heat transport across 60N, is responsible for the dispersal of surface drawn energy in sea ice retreat regions and maintaining the air-sea temperature gradient that enhances surface turbulent fluxes. Burt et al. [2016] provide evidence for such an atmospheric circulation response where reduced fall/winter Arctic Ocean sea ice induces a thermal contrast with the colder Arctic continents that drives winds that promote stronger surface turbulent fluxes. An alternate explanation is that reduced sea ice and enhanced surface turbulent fluxes lower local atmosphere pressure and influence the Arctic circulation and the manner in which energy is redistributed from sea ice retreat regions Arctic-wide [Bengtsson et al. 2004]. Therefore, understanding the inter-model differences in this atmospheric response to increased surface turbulent fluxes in sea ice retreat regions appears critical to improving projections of Artic warming.

Further support for the importance of sea ice retreat regions in driving Arctic Amplification is gathered by contrasting energy changes in sea ice retreat regions vs. ice-free ocean regions. Sea ice free ocean regions show much less warming and a surface turbulent flux increase into the surface in these regions due to a decreased air-sea temperature gradient. In ice-covered regions, such as the central Arctic, the overall energy flows are the similar to sea ice retreat regions but exhibit much smaller changes because sea ice has a lower heat capacity and weaker fall/winter surface turbulent fluxes. Similar amount of warming due to LWCS is found in both of sea ice covered and sea ice retreat regions.

A complicating factor here is the influence of the ocean mixed layer depth and dynamics in modulating the seasonal heat transfer. A deeper ocean mixed layer stores more energy in summer, acts to delay fall sea ice freeze-up, can draw heat from deeper depths in the ocean, and contributes to larger surface turbulent fluxes. Because of the relationship between seasonal energy transfer and Arctic Amplification we hypothesized that the ocean mixed layer depth would influence the amount of warming. The eight CMIP5 models that have archived ocean mixed layer depth have been analyzed showing very large inter-model differences. The average ocean mixed layer depth in the Barents-Kara Seas region ranges from 13 to 95 meters in fall. Our analysis shows no correlation between the control climate mixed layer or its changes with warming with Arctic Amplification. Despite the lack of correlation, this range of ocean mixed layer depth significantly influences the ability of the ocean to store energy and modulate surface turbulent fluxes, sea ice variability, and the atmospheric circulation. Therefore, an improved understanding of the causes and consequences of the inter-model spread in ocean mixed layer depth and the sensitivity of sea ice and surface turbulent fluxes to changes in ocean mixed layer depth is needed.

This discussion is included in the text.

3. The authors argue that Delta OHF could be an observational constraint for AA – however, for that to be of practical relevance, it would have to be demonstrated that the signal in Delta OHF emerges earlier than that in Arctic-wide temperature change.

We agree that an observational constrain would be best if it emerged before warming occurred. However, this is not required for such a constraint to be useful. Given the relationship between delta_OHF and AA over the next 20 years (or previous 20 years using reanalysis), the relationship that we demonstrate here provides an indication of how much warming we should expect over the subsequent 20 year and longer. This relationship links seasonal energy transfer to the efficiency of AA, therefore models can produce similar warming with different changes in the seasonal energy transfer. Therefore, this relationship is useful as an observational constraint.

Minor issues:

1. title: I would suggest referring to the inter-model spread here.

The word inter-model spread has been added to the title.

2. ll. 55 ff. Maybe cite earlier work on the albedo effect, too (Budyko, Arrhenius).

Added reference on the earlier work on the surface albedo effect

3. l. 79. I believe there is a plot on the seasonal cycle in Pithan and Mauritsen 2014.

We have revisited this paper and yes there is a plot that looks at the summer vs. winter feedback differences. This plot does not resolve the important energy changes that occur over the entire seasonal cycle, however. We have revised this text to be consistent with what they show.

4. l. 103 ff. Should it be considered that zonal means near the pole are an average over a much smaller area than at lower latitudes?

This is a consideration that we feel is understood and very infrequently stated in similar research.

5. l. 149 Graversen

Fixed.

6. table 1: I suggest naming the institute rather than the country, and maybe referring to the atmospheric model in use, too (to make model similarities more transparent).

The table has been revised and is not included in supplementary material.

Reviewer #2 (Remarks to the Author):

The paper is attempting to assess contributions of various processes to polar amplification based on a host of CMIP5 models. An approach somewhat similar to one suggested my Ming Cai in his

2005 paper is utilized. Analysis suggests that the 'hotspot' that contributes the most to the warming in the Arctic is roughly collocated with the Barents-Kara seas. This is not a new result, which is one of the reasons why the paper should be rejected. The analysis raises certain questions as well (see below). The paper is difficult to read and follow: too many acronyms, the text flow is not smooth and sometimes it is hard to see the logic behind authors' arguments. I suggest rejecting the paper. Below are my more detailed comments.

We have taken all of the comments and improved the presentation and clarity of the manuscript. We think that our new analysis and discussion is worthy of publication. Moreover, we agree that previous work has indicated the Barents-Kara Seas regions as a ‘hotspot’ of Arctic warming. However, our work presents a more complete process loop connecting this region (and other sea ice retreat regions) to Arctic Amplification. The two new aspects of our work explaining the inter-model spread are (1) the tie between a local surface turbulent fluxes and Arctic wide LWCS via an atmospheric circulation response and (2) the large inter-model spread in ocean mixed layer depth influences sea ice and surface turbulent flux yet does not correspond to the inter-model spread in AA.

How does the utilized 'feedback analysis' relate to the classical approach described in Peixoto Oort "Physics of Climate"? All I can see is the analysis of the surface heat budget. This is actually an extremely poor approximation to M.Cai's approach described in his 2005 paper. No dynamics of any kind is included. Non-locality of any kind can not be described IN PRINCIPLE using a local surface heat budget equation.

What the authors did is the following. They calculated all the surface fluxes in the 'present day' and future climates and claim that changes in the fluxes can serve to explain various feedbacks. The fundamental question here is whether the fluxes can be considered as forcings affecting the climate or they are a part of the climate system responding to changes. Do they form independently of the state of the climate? I would argue they do not. Therefore they cannot be separated ino a special category and/or be considered as forcings independent of the response (or the change of the system). The whole approach needs to be justified on a much more solid basis.

Our analysis technique is a surface energy budget decomposition and is not a complete feedback analysis approach. Our approach is consistent with the conventional TOA feedback analysis approaches analyzing feedbacks through a decomposition of radiative flux changes due to various terms (e.g., water vapor, clouds, albedo, etc.) such as Pithan and Mauritsen [2014]. Our approach does not do a complete decomposition of the various feedbacks. The method of decomposition the temperature change into the partial temperature contributions as presented in Lu and Cai [2009] essentially divides each energy budget term by the Planck Feedback. This approach rewrites the surface energy budget term contributions to the surface temperature change into temperature units, which are more intuitive. The approach provides the temperature change that occurs when only considering the radiative response (e.g., Planck Feedback).

The reviewer accurately describes our approach. From our analysis we are unable to separate whether the changes in the surface energy budget are forcing or feedbacks. This is true of conventional feedback analysis techniques as well. In our view, this is irrelevant to the understanding we are trying to gain. Specifically, what terms and processes are driving the inter-

model differences between Arctic warming produced in CMIP5 models. Our approach is justified because the changes in the surface energy budget control the surface temperature and sea ice responses in the Arctic. Understanding the drivers of the inter-model differences in the surface energy budget provides insight into how we can improve the models.

We do not go as far as Cai [2005] in our analysis and this was never intended. We now include an assessment of the atmospheric poleward heat transport (energetically constrained) to indicate contributions from non-local changes.

What do the authors mean by regional in the title? If the main 'hotspot' is in the Barents-Kara seas, how does that signal spread all over in the entire Arctic? How does the ocean contribute to forming the 'hotspot'? How local is this contribution from the ocean? Does the North Atlantic play a role and if it does, how local is the signal in the Barents-Kara seas?

The meaning of regional in the title is that it appears changes in specific regions, as opposed to Arctic-wide, that explain the inter-model differences. The signal is spread throughout the Arctic by the atmospheric circulation and it is the differences in the circulation responses between models that we hypothesize enable the increases in air-sea temperature gradient [e.g., Burt et al. 2016].

Indeed, the ocean plays a role through ocean mixed layer processes and through ocean heat transport. As we mention in the manuscript, ocean heat transport is important but cannot be assessed on its own here. However, Laine et al. [2016] demonstrates that ocean heat transport plays a very small role in CMIP5 model projected Arctic warming by 2100.

What are those 'interactions' in the title? It is not clear what the authors mean.

Interactions in the title refers to the seasonal exchange of energy from the summertime storage to the fall/winter surface turbulent flux release facilitated by a delayed fall freeze-up and enhanced air-sea temperature gradient. We have revised the title.

Some deeper background research of the topic is recommended. A number of papers (even before any of M.Cai's papers) that actually describe and explain polar amplification mechanisms should have been included in the introduction. The Graversen et al (2008) Nature paper cannot be used as the one that introduced the arctic (or polar) amplification. Besides, it is also well known that the analysis described in that paper is not quite free of flaws. Note that R.G.Graversen's name is misspelled (line 65).

Arrhenius S (1896) On the influence of carbonic acid in the air upon the temperature of the ground. Phil Mag 41:237–276

Flannery BP (1984) Energy-balance models incorporating transport of thermal and latent energy. J Atm Sci 41:414–421

Schneider EK, Lindzen RS, Kirtman BP (1997) A tropical influence on global climate. J Atm Sci

54:1349–1358

Rodgers KB, Lohmann G, Lorenz S, Schneider R, Henderson GM (2003) A tropical mechanism for Northern Hemisphere deglaciation. Geochem Geophys Geosyst 4(5):1046. DOI:10.1029/2003GC000508

Alexeev VA, Langen PL, Bates JR (2005) Polar amplification on surface warming on an aquaplanet in ‘‘ghost forcing’’ experiments without sea ice feedbacks. Clim Dyn. doi:10.1007/s00382-005-0018-3

We have included these references.

Reviewer #3 (Remarks to the Author):

Review of the manuscript entitled “Projected Arctic warming controlled by regional atmosphere-ocean interactions” by R.C. Boeke and P.C. Taylor

Research aimed at an assessment of relative contribution of individual feedbacks to Arctic Amplification (AA) is important and timely. However, I’m concerned with the applicability of CMIP5 model simulations for the pan-Arctic region to advance it. Specifically, given the relatively coarse spatial and temporal model resolution of CMIP5 model components over the Arctic, I remain skeptical about their usefulness to assess the relative impact of processes and feedbacks on the magnitude of AA. Especially, when the primary diagnostics for those contributions and impacts is the inter-model spread in simulated AA, as elaborated below.

We agree with your comment about the utility of models and specifically the CMIP5 ensemble to assess the specific mechanisms generating AA. While in the beginning of this project we attempted to perform such a feat, we quickly realized that this is not really possible. As such, our analysis focuses on the processes that explain the inter-model spread in AA. The motivation for this research is that projections of the Arctic’s future are made with this ensemble and similar ensembles in the future. We must understand the differences between these model projections in order to narrow the uncertainty in model projections or at the least be able to assess whether the spread between models is due to natural variability or differences in their forced responses. This can only be accomplished using CMIP5 or a comparable model ensemble projecting future changes in AA.

Moreover, I find it troubling that the term in the surface energy budget, the ocean heat storage and transport, critical to the conclusions of this paper is not calculated directly from model output but estimated as a residual from the surface energy imbalance. This makes it impossible to understand and assess the relative importance of specific processes and feedbacks controlling the ocean heat storage and transport and their contribution to AA. While the authors provide the justification for their approach, i.e. the lack of proper CMIP5 model output, the problem remains that they conclude on the importance of the term, which is not directly calculated from models, nor can be directly evaluated for its contribution to the model spread in simulating AA.

We would also like to include a direct calculation of all of the terms. And, we hope that future CMIP activities will enable direct calculations. However, for a smaller subset of models (eight) we were able to assess the influences of ocean mixed layer depth. The results are interesting and puzzling at the same time. First, we find a very significant spread in the ocean mixed layer depth in the Barents-Kara Seas regions (13 to 85 meters in fall) and that this spread does not appear to correspond to more or less AA. We hypothesized that the ocean mixed layer depth would be a key piece of this puzzle because the seasonal energy transfer is modulated by the ocean mixed layer depth, however our results show no relationship. We think that ocean mixed layer depth and dynamics are still critical here because the ocean is an important reservoir of heat influencing sea ice, surface turbulent fluxes, and the atmospheric circulation. Moreover, the lack of correlation may indicate a bias in the model representation of OML dynamics, such as the known bias in upper ocean vertical mixing [Huang et al. 2014].

Also, I find the concept of the Arctic Amplification Index and results shown in Fig.5 overly complicated and hard to interpret physically. Given that these results are critical to the main conclusions of this paper, I believe they could be either better explained or presented by alternative means, easier for physical interpretation. In particular, the inset maps of the annual and seasonal standard deviation among models for each AAI in Fig.5 show largest magnitudes of HSTOR and HFLUX in the Greenland Sea throughout the year and also in the Labrador Sea during winter (JF), which is not discussed in the text and different from the spatial pattern of the inter-model standard deviation of projected surface temperature change shown in Fig.3e-h

This concept has been removed and we now only discuss the partial temperature contributions and their inter-model spread. We agree that this concept was confusing and is not necessary.

Given the importance of changes in the sea ice cover to AA, I argue it's critical to show how CMIP5 models represent it (preferably using ice thickness distribution) for the first and last 20 years of simulation. One key reason for that is to demonstrate model skill in representing the present time spatial distribution of sea ice and its change by 2100, which will not only affect AA through local feedbacks but will also reflect on model biases and spread. For example, many if not all CMIP5 models, likely experience similar biases in representing northward oceanic heat transport from the Pacific through (narrow and shallow) Bering Strait, which should translate onto their simulation of sea ice distribution in the western Arctic but not be necessarily revealed in the model spread.

We agree that sea ice thickness is a critical consideration but have decided not to include it in the paper. Sea ice thickness is important to the energy flows we discuss, however sea ice concentration is more important to the seasonal changes in ocean heat storage. We have added the model projections of sea ice concentration to Fig. 2. Models certainly exhibit biases in sea ice due to ocean heat transport, however our analysis indicates that no correlation between the model mean sea ice cover in the Barents-Kara Seas region and AA. In other words, models that begin with more sea ice in the Barents-Kara Seas region do not warm more or less than models that begin with less sea ice.

In summary, I find this manuscript addressing an important scientific and societal problem and worth publication in Nature Communications, subject to major revision in order to address the

main concerns above. An extended discussion of the limitations of CMIP5 models in representing processes and feedbacks of relevance to AA, including those controlling the two terms, HSTOR and HFLUX, would be a critical part of this revision.

We have included a number of additional references and discussion to the process contributions and limitations of CMIP5 models. As noted above, we have included additional analysis specifically of the ocean mixed layer depth and its relationship with sea ice and surface turbulent fluxes. Indicating a significant sensitivity but that does not relate to the inter-model spread in AA.

Detailed comments:

- L.27-32: How well the ocean heat storage and mixed layer processes as well as surface turbulent fluxes and air-sea interactions are represented in the CMIP5 climate models?

Large discrepancies exist in the surface turbulent fluxes in CMIP5 climate models (Taylor et al. 2018) and in the ocean mixed layer depths.

- L.34-35: what evidence exists or can be provided to support the 2nd conclusion?

The evidence for this conclusion now comes from Fig. 9 showing the significant relationships between ocean mixed layer depth, surface turbulent fluxes, and sea ice cover. While there is no correlation with AA, the fact that the models show such a large difference in these relationships indicates that the representation of these interactions needs to be improved in models.

- L.109-112 and Fig. 3: explain and justify the criteria for defining seasons as in Fig. 3, specifically the sunlit (MAMJJAS) season?

The primary reason to split seasons in this manner is that this categorization separates the analysis into months when the surface albedo feedback is active and months when it is not.

- L.112-118: explain what observational data have been used to constrain the model spatial patterns and magnitudes f AA against reality?

We have not used any observational data to constrain the model spatial patterns. This analysis is focused on trying to understand why and how the models differ.

- L.116-121: clarify the contradicting statements about the warming in the Norwegian Sea in these two sentences;

This has been clarified.

- L.120-121: clarify/define what ‘heat transport from lower latitudes’ is referred to here and present or discuss model results on those transports;

This phrase has been removed.

- L.126: discuss measures / metrics to support the statement about the SEB being more relevant to the state of the Arctic system, compared to the TOA energy budget;

We have change this statement to read “Arctic surface temperature” instead of “state of the Arctic system” instead of adding further discussion.

- L.132: please consider changing the name of HSTOR from “surface” to “ocean” heat storage and transport, since the surface is just an interface so it can’t store or transport properties.

We have changed the name to ocean heat storage.

Reviewer #4 (Remarks to the Author):

Review of “Projected Arctic warming controlled by regional atmosphere-ocean interactions” by Boeke and Taylor.

General
This is nice and informative paper on the reasons of the intermodel differences in Arctic Warming (AW). This, they show, can be attributed to regional surface heat fluxes. I believe this is a new(ish) insight (see comments below), and the paper is very well written, and I have very little comments on their analyses of the models, which seem sound and complete. That being said, I have a couple of other specific comments, which should be addressed before the paper would be, in my view, suitable for publication.

Specifics
1. Roughly the first half of the paper (Figs. 1-3) reads as an introduction, or even a repetition of results long known. In fact, on line 155 they even state that the results presented here, based on CMIP5 models, basically replicate those of Lu/Cai, 2009, which were based on CMIP3 models. While reading this first half, I got the impressing that this part can be significantly shortened with some proper referencing. Even though the first half reads very well, it is also evoking thoughts as “when does the new stuff start”, at least with me. Hence, I strongly suggest to shorten this part a great deal.

We have revised and shortened the introduction of the paper.

2. While I understand that the paper focuses on intermodel differences, it would still be nice to have the model-mean somewhere as a line or value in the various figures/tables, if only for readers to see how far a particular model if ‘off’ the model mean.

Ensemble mean values have been added to several figures.

3. In Fig. 7, one of the 3 maps in panel a seems to be missing.

Figure 7 has been changed.

4. One critically missing issue relates to sea ice. Obviously, the huge changes in the BK seas in surface heat flux and storage are related to sea ice retreat (and thinning), and the intermodel differences in the surface heat fluxes are therefore related to those in sea ice retreat. The authors are well aware of this, I would assume, and it is indeed mentioned, but only, seemingly, as a side note. I believe sea ice retreat is crucial to the processes at hand, and should be discussed (and shown!!) much more prominently. One would think that models with the greatest changes in surface heat flux would also exhibit the largest sea ice retreat (and thinning), related to greater warming…

This is true, the delay in fall sea ice freeze-up is at the center of our explanation. We have significantly expanded upon the discussion of AA processes and the sources of inter-model spread in the revised manuscript. The relationship between sea ice and surface turbulent fluxes is illustrated now in Fig. 8. Moreover, we now show the inter-model differences in the projected changes in sea ice concentration in Fig. 2.

5. Related to 4… The authors state that changes in surface heat fluxes (in the BK seas) control warming, but how are they so sure of the causality? Models that for some reason exhibit greater AW also have stronger sea ice retreat, and thus bigger changes in surface heat fluxes. So the changes in the heat fluxes may equally well be the result of AW, not the cause (or both). More analyses is required to demonstrate the hypothesized causality, perhaps by analysing leads/lags.

Causality is always a tricky part of inter-model analyses such as the one presented here. First, you are correct and that the causality of our mechanisms is not assured. However, we have added discussion, references, and further analysis that supports causality. First, the inclusion of the scatterplots (Fig. 7) directly attributing the changes in the seasonal transfer of energy are primarily due to summer surface albedo feedback and fall/winter surface turbulent fluxes. Moreover, we show that these change on focused in sea ice retreat regions and describe that this relationship is opposite in ice-free ocean regions (e.g., North Atlantic Ocean) and much weaker in sea ice cover regions (e.g., central Arctic Ocean). These results demonstrate that if it weren't for the sea ice retreat regions Arctic Amplification would be much weaker and, in some cases, non-existent. Further, Fig. 7 illustrates that changes in atmospheric poleward heat transport are not driving inter-model differences in AA but are dampening the inter-model spread. This result does not imply that increased LW downwelling radiation to the surface due to changes in poleward heat transport are not important for driving AA, but implied that they do not explain the inter-model spread.

6. The authors seem to have overlooked recent research that relates variations in surface heat fluxes in the BK region to Arctic Decadal Variability (ADV), see e.g. http://onlinelibrary.wiley.com/doi/10.1002/2016JD026058/full. The finding that the surface fluxes are key in ADV may relate to the issue presented here in the sense that the atmosphere-ocean interactions in the BK area are crucially important for Arctic climate, so for both AW and variability. This would not surprise me at all. I think this link (equivalent processes being crucial for AW and variability) is worth looking into.

Thank you for this recommendation.

Reviewer #4 (Remarks to the Author):

The authors have addressed my comments in a satisfactory manner. I recommend publication.
