# Supplementary material for: Seasonal energy exchange in sea ice retreat regions contributes to differences in projected Arctic warming
Source: Nat Commun. 2018 Nov 27;9:5017. doi: 10.1038/s41467-018-07061-9 (PMC6258731; doi:10.1038/s41467-018-07061-9)
Supplement: Supplementary file 1 — Peer Review File [file 41467_2018_7061_MOESM1_ESM.pdf]

Supplementary Information

Seasonal energy exchange in sea ice retreat regions contributes to differences in projected Arctic warming

Robyn C. Boeke

Science Systems Applications Inc., Hampton, Virginia

Patrick C. Taylor*

NASA Langley Research Center, Climate Science Branch, Hampton, Virginia

*Corresponding author address: Patrick C. Taylor, NASA Langley Research Center, 21 Langley Blvd. MS:420, Hampton, VA, 23681 (patrick.c.taylor@nasa.gov)

Table/Figure Legends

Supplementary Table 1. List of CMIP5 models used in this study and their country of origin.

Supplementary Figure 1. Map of Arctic regions discussed in this paper.

Model	Institution
ACCESS1.0	Commonwealth Scientific and Industrial Research Organisation, Bureau of Meteorology
ACCESS1.3	Commonwealth Scientific and Industrial Research Organisation, Bureau of Meteorology
BCC-CSM1.1	Beijing Climate Center
BCC-CSM1.1(m)	Beijing Climate Center
BNU-ESM	College of Global Change and Earth System Science, Beijing Normal University
CanESM2	Canadian Centre for Climate Modelling and Analysis
CCSM4	National Center for Atmospheric Research
CESM1-BGC	National Science Foundation, Dept. of Energy, National Center for Atmospheric Research
CESM1-CAM5	National Science Foundation, Dept. of Energy, National Center for Atmospheric Research
CNRM-CM5	Centre National de Recherches Meteorologiques, Centre Europeen de Recherche et Formation Avancees en Calcul Scientifique
CSIRO-Mk3.6.0	Commonwealth Scientific and Industrial Research Organisation in collaboration with the Queensland Climate Change Centre of Excellence
GFDL-CM3	Geophysical Fluid Dynamics Laboratory
GISS-E2-H	NASA Goddard
GISS-E2-R	NASA Goddard
INM-CM4	Institute for Numerical Mathematics
IPSL-CM5A-LR	Institut Pierre-Simon Laplace
IPSL-CM5A-MR	Institut Pierre-Simon Laplace
MIROC5	Atmosphere and Ocean Research Institute (The University of Tokyo), National Institute for Environmental Studies, Japan Agency for Marine-Earth Science and Technology
MPI-ESM-MR	Max Planck Institute for Meteorology
MPI-ESM-LR	Max Planck Institute for Meteorology
MRI-CGCM3	Meteorological Research Institute
NorESM1-M	Norwegian Climate Centre
NorESM1-ME	Norwegian Climate Centre

Supplementary Table 1. List of CMIP5 models used in this study and institution

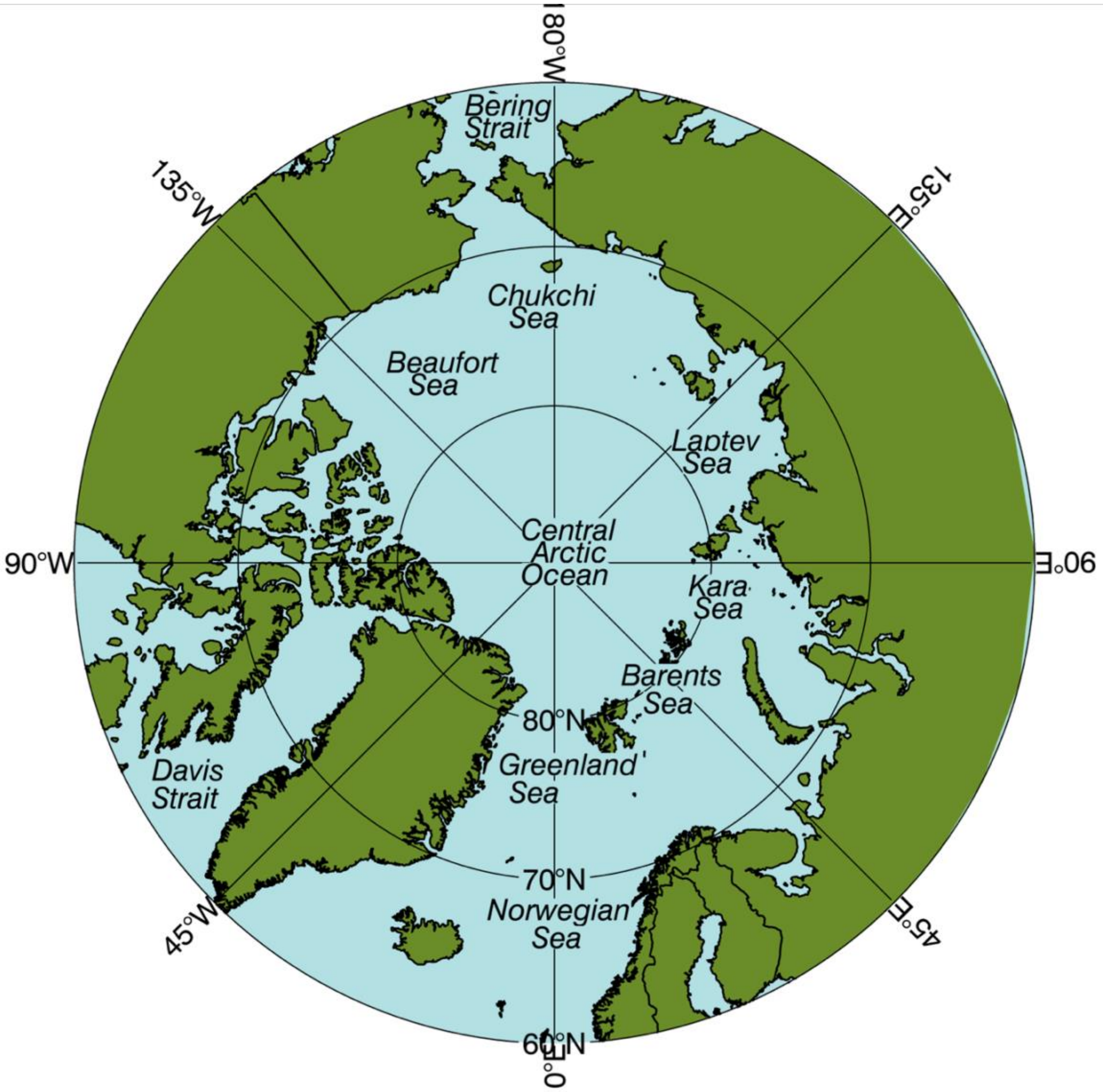

Supplementary Figure 1. Arctic map and defined regions.
